# 1388. Seasonality of Microbiology of Combat-related Wound Infections in Afghanistan

**DOI:** 10.1093/ofid/ofac492.1217

**Published:** 2022-12-15

**Authors:** Matthew A Soderstrom, Dana M Blyth, Leigh Carson, Wesley R Campbell, Joseph Yabes, Faraz Shaikh, Laveta Stewart, David R Tribble, Clinton K Murray, John L Kiley

**Affiliations:** SAUSHEC, san antonio, Texas; Walter Reed National Military Medical Center, Bethesda, MD; Infectious Disease Clinical Research Program, Bethesda, Maryland; Walter Reed National Military Medical Center, Bethesda, MD; Brooke Army Medical Center, San Antonio, Texas; Infectious Disease Clinical Research Program, Bethesda, Maryland; Infectious Disease Clinical Research Program, Bethesda, Maryland; Uniformed Services University of the Health Sciences, Bethesda, Maryland; Brooke Army Medical Center, San Antonio, Texas; Brooke Army Medical Center, San Antonio, Texas

## Abstract

**Background:**

We examined the seasonality of wounds and wound infections, including occurrence of multidrug resistance, among combat casualties injured in Afghanistan.

**Methods:**

The Trauma Infectious Disease Outcomes Study is a retrospective observational study of infectious complications among military personnel wounded during deployment (06/09-12/14). Wound cultures obtained ≤7 days following injury in Afghanistan were assessed. Epidemiologic, clinical, and microbiologic data were analyzed by injury season [winter (1 Dec-28/29 Feb), spring (1 Mar-31 May), summer (1 Jun-31 Aug), and fall (1 Sep-30 Nov)]. Multidrug-resistant (MDR) determinations for Gram-negative and Gram-positive organisms were per standardized definitions.

**Results:**

The study population included 316 patients with a median of 3.5 (IQR 3-5) days from injury to initial culture. Gram-negatives (N=188, 59.5%) were more commonly isolated from wound cultures in summer (N=81, 43.1%) and fall (N=57, 30.3%) versus winter (N=18, 9.6%) and spring (N=32, 17%) (p< 0.001). The MDR Gram-negatives (N=69, 21.8%) were more common in summer (N=26, 37.7%), and fall (N=26, 37.7%) versus winter (N=3, 4.3%) and spring (N=14, 20.3%) (p=0.028). Wound infections were diagnosed in 198 (63%) patients. The pattern for infecting Gram-negative isolates (N=143, 72.2%, **Table 1**) was similar to that of overall Gram-negative isolates: summer (79.5%) and fall (83.6%; p< 0.001); MDR Gram-negatives (summer, 25.6%) and (fall, 41.8%; p=0.015). *Escherichia coli* and *Enterobacter* spp. were the most common infecting Gram-negative bacilli with no significant difference across the seasons. There was a higher proportion of infecting *Acinetobacter baumannii* isolates in the summer and fall compared to winter and spring. Infecting Gram-positive isolates (N=128, 65%) were not significantly different by season. Anaerobes associated with infections were also identified (N=30, 15%) with a higher proportion in the winter compared to summer, fall, and spring (p=0.036).

**Figure:**
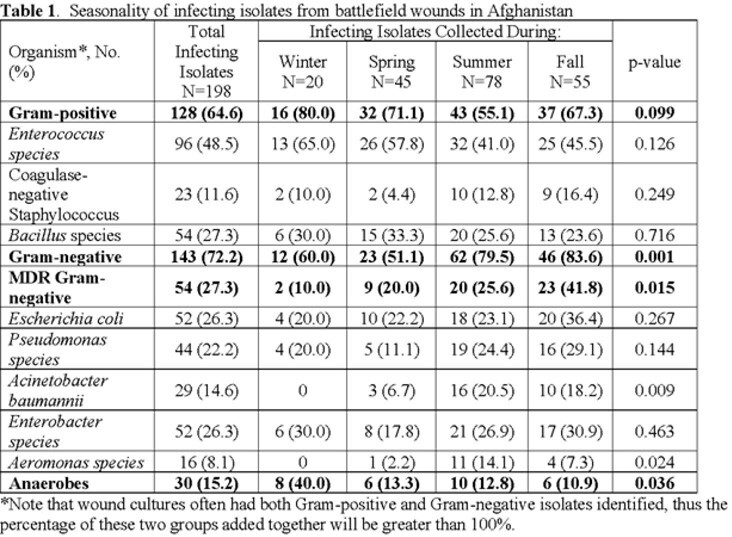

**Conclusion:**

Gram-negatives, including MDR Gram-negative infecting organisms, were more common in summer/fall months in service members injured in Afghanistan. This may have implications for empiric antibiotic coverage during these months.

**Disclosures:**

**David R. Tribble, DrPH**, AstraZeneca: The HJF, in support of the USU IDCRP, was funded to conduct or augment unrelated Phase III Mab and vaccine trials as part of US Govt. COVID19 response.

